# Mr. Mitchell's Case of Wound in the Lungs

**Published:** 1826-07

**Authors:** 


### CASE OF WOUND IN THE LUNGS.*

A marine of a plethoric habit, was severely wounded in the chest, by a

* Related by James Mitchell, Surgeon, Royal Navy, Wooler. Ed. Journal, April, 1826.

cannister shot. When seen the day after, he was found sitting on a pallet, the head bent forwards, mouth open, and gasping for breath, face swollen and much flushed, and the pulse full and bounding. Two pints of blood were drawn, which enabled him to speak and breathe more freely. On examination, the shot was found to have passed through the right breast below the nipple, and to project in the back through a wound it had made under the right scapula. When extracted, a large quantity of frothy blood issued out, and a lighted candle, held to the wound, was instantly extinguished. The wound was cleaned and dressed, and the patient laid on his back, after which he discharged by the mouth, a large quantity of frothy blood and mucus from the lungs. In an hour, V. S. ad ℔ j., cool subacid drinks and sulphate of magnesia, with strict abstinence, were employed. He took four times a day, *t. digital. gtt.* x. acid. sulph. dilut. gtt. xv. liq. plumb. acetat. gtt. iij. aq. font. ℥i. ℳ. ft. haust. and lost, in all, by bleeding, eight pounds of blood, which relieved the constitutional and local irritation so much, that he could lie down with ease, and breathe comparatively freely. His bowels were opened by the salts, and he took the following anodyne; ℞. Tinct. op. gtt. xxx. vin. antim. ʒi. spt. æth. nitros. ʒi. aq. ℥i. ℳ. ft. haust.

He spent a pretty tranquil night, but towards morning he had an increase of the fever, hæmoptysis and dyspnœa, which were dissipated by V. S. ad ℔i. Treatment as before. During the day he had two attacks, which were each relieved by the abstraction of a pound of blood. Anodyne at night.

Third day: much better. From this time he continued to improve daily, and required no further bleeding. The bowels were kept open; the circulation restrained by sedatives and abstinence, and in five weeks he was completely cured, having only a little difficulty of breathing on walking quickly or up an ascent.

*Remarks.* This man had a very fortunate escape, almost as fortunate as Mr. Maiden's patient, who recovered after the shaft of a gig had passed through the lungs, and pinned him to the wall.

---

